# Hesitance and Misconceptions about the Annual Influenza Vaccine among the Saudi Population Post-COVID-19

**DOI:** 10.3390/vaccines11101595

**Published:** 2023-10-15

**Authors:** Baraa Alghalyini, Tala Garatli, Reela Hamoor, Linda Ibrahim, Yara Elmehallawy, Dima Hamze, Zain Abbara, Abdul Rehman Zia Zaidi

**Affiliations:** Department of Family & Community Medicine, College of Medicine, Alfaisal University, Riyadh 11533, Saudi Arabia; balghalyini@alfaisal.edu (B.A.); tgaratli@alfaisal.edu (T.G.); rhamoor@alfaisal.edu (R.H.); librahim@alfaisal.edu (L.I.); yelmehallawy@alfaisal.edu (Y.E.); dhamze@alfaisal.edu (D.H.); zabbara@alfaisal.edu (Z.A.)

**Keywords:** influenza vaccines, vaccine hesitancy, misconceptions, health knowledge, attitudes, practice, cross-sectional studies, influenza, human, health promotion, vaccination, public health, Saudi Arabia

## Abstract

(1) Background: Vaccination is a cornerstone of public health strategy for mitigating the morbidity and mortality associated with seasonal influenza. However, vaccine hesitancy and misconceptions pose significant barriers to this effort, particularly in the context of the coronavirus disease 2019 (COVID-19) pandemic. This study aimed to investigate the transfer of COVID-19 vaccine hesitancy to the influenza vaccine and to identify misconceptions about the influenza vaccine among the Saudi population in the post-COVID-19 era. (2) Methods: A web-based, cross-sectional study was conducted between February and June 2023 using a questionnaire adapted from the Adult Vaccine Hesitancy Scale (aVHS). The questionnaire was disseminated to 589 Saudi residents, aged 18 and above, with access to digital devices. Data were analyzed via logistic regression analysis to determine the associations between vaccine hesitancy, knowledge of influenza, and baseline characteristics. (3) Results: This study found that 37.7% of respondents exhibited vaccine hesitancy, while 56.7% demonstrated good knowledge about influenza. There was a significant relationship between nationality and vaccine hesitancy (*p*-value > 0.05), with non-Saudi respondents exhibiting higher hesitancy. Logistic regression analysis revealed significant associations between vaccine hesitancy, age, and nationality. Meanwhile, participants with higher educational qualifications showed greater knowledge about influenza. (4) Conclusions: The findings highlight an important crossover of COVID-19 vaccine hesitancy to influenza vaccines. This study underscores the need for targeted public health interventions to address misconceptions about the influenza vaccine, particularly among certain demographic groups, in order to improve influenza vaccine uptake in the post-COVID era.

## 1. Introduction

Seasonal influenza, colloquially known as the flu, is a recurring acute respiratory ailment attributed to a spectrum of influenza viruses. It is categorized into four distinct types: A, B, C, and D. Predominantly, types A and B are implicated in annual outbreaks, and they have been substantially associated with seasonal flu morbidity and mortality [1]. Data from the Center for Disease Control and Prevention (CDC) substantiated the rapid transmissibility of the virus, indicating its role in approximately nine million infections and 5000 consequential deaths during the 2021–2022 cycle worldwide [2]. Such a pervasive spread underscores the need for effective prevention measures. To this end, the seasonal influenza vaccine emerged as the most potent preventive measure, providing optimal protection against associated morbidities [1].

However, globally, and notably in regions like Saudi Arabia, there has been discernible reluctance toward vaccine uptake. This hesitancy is augmented by prevailing misconceptions, thus posing a potential health risk to susceptible populations such as pregnant women, the immunocompromised, and the elderly [1]. A myriad of studies from Saudi Arabia and its neighboring Gulf States have identified these barriers and their underlying causes [3,4,5,6,7,8].

The advent of the coronavirus disease 2019 (COVID-19) pandemic in 2020, caused by the severe acute respiratory syndrome coronavirus 2 (SARS-CoV-2), introduced additional complexities in public health messaging concerning vaccinations. With over 760 million confirmed cases globally by 26 April 2023, of which 800,000 were reported in Saudi Arabia, the pandemic, which was declared official by the World Health Organization (WHO) in March 2020, engendered pressing concerns [9,10]. The expeditious development and deployment of COVID-19 vaccines accentuated apprehensions about vaccine safety and efficacy. A 2021 study corroborated these apprehensions by revealing that only 48% of Saudi adults expressed willingness to receive the COVID-19 vaccine [11]. Such hesitancy has significant implications as, given the pivotal role vaccines play in reducing hospitalization and mortality rates, reduced vaccination rates would subsequently increase the economic burdens on healthcare systems. In the recent literature, such as Minshawi et al., there were suggestions that the hesitancy exhibited toward the COVID-19 vaccine might have a spillover effect on attitudes toward the influenza vaccine [12]. Given this context, our study endeavors to elucidate the potential transference of COVID-19 vaccine hesitancy onto the influenza vaccine and to discern the associated misconceptions among the Saudi population in the post-COVID-19 era.

## 2. Materials and Methods

### 2.1. Study Design and Sampling Strategy

An anonymous web-based, cross-sectional study was conducted from February 2023 to June 2023. The questionnaire used was adapted from a previously validated questionnaire, the “Adult Vaccine hesitancy scale (aVHS)” [13], and combined with a survey that was utilized in a study approved by an institutional review board of King Fahad Medical City in Riyadh, Saudi Arabia [14], to collect data from a sample of 500 participants in Saudi Arabia. The questionnaire included both open-ended and closed-ended questions. The survey was disseminated through various online platforms and social media channels that are prevalent in Saudi Arabia to ensure a diverse range of respondents. The study did not conduct a separate pilot because the questionnaire was based on previously validated scales, and it was refined with the expertise of the co-authors. The survey was open to all Saudi Arabia residents aged 18 years and above. All adults equipped with cellphones or personal computers had the capability to participate in the survey. Implicit consent was assumed upon participation, with respondents aware that their engagement was entirely voluntary.

### 2.2. Sample Size and Sampling Technique

In this study, a two-sided test with a 95% confidence interval and a power of 80% was used. Based on previous studies on vaccine hesitancy, it was assumed that the prevalence of vaccine hesitancy in the Saudi population post-COVID would be around 50%. Using these assumptions, the following formula for sample size calculation for a cross-sectional study was used:

n = (Z^2^ × P × (1 − P))/(d^2^)

where n is the sample size, Z is the standard normal deviation corresponding to the level of confidence, P is the expected proportion of vaccine hesitancy, and d is the margin of error.

Based on this formula, it was calculated that a sample size of 384 would be needed to achieve a power of 80% with a margin of error of 5%. However, to account for potential non-responses or incomplete surveys, the sample size was increased to 500.

In summary, the sample size of 500 participants was determined based on power analysis, using a two-sided test with a 95% confidence interval and a power of 80% and assuming a prevalence of vaccine hesitancy of 50%. This sample size calculation provided sufficient statistical power to detect meaningful differences and relationships between variables, thus ensuring the validity and reliability of the study findings.

### 2.3. Study Outcomes and Variables

An online survey was developed using Google Forms to ensure respondent anonymity. This survey was composed of several questions designed to support the primary objectives of the research. Included within the questionnaire were structured queries, specifically those related to participant demographics. An introductory section of the research instrument delineated the aims of the study, reassured respondents of the confidentiality of their submissions, and granted them the autonomy to abstain from responding to specific items or to retract their participation entirely. Moreover, the aims, methodologies, potential risks, and benefits of the investigation were lucidly articulated to the participants. Before participating, respondents confirmed their informed consent. All collected data were securely housed, with exclusive access granted only to the research team, thereby ensuring protection against unauthorized use, access, or dissemination. The principal investigator guaranteed the confidentiality of the data. A few of the close-ended questions examined demographic data, including age, gender, education, and occupation, as well as opinions regarding vaccination, vaccine hesitancy, vaccine effectiveness and safety, and healthcare system trust. Participants were given the opportunity to express their opinions and views about the influenza vaccine, as well as any misconceptions they might have, through the presence of open-ended questions.

### 2.4. Statistical Analysis

Data analysis was conducted using the Statistical Package for the Social Sciences (SPSS), version 28. Continuous data were represented in terms of the mean and standard deviation, while categorical data were depicted as frequencies and percentages. To assess the data distribution, both the Kolmogorov–Smirnov and Shapiro–Wilk tests were executed. The validity of the scales employed was ascertained by evaluating their Cronbach’s Alpha, which demonstrated high internal consistencies: the Adult Vaccine Hesitancy Scale registered at 0.869, while the Knowledge of Influenza Scale arrived at 0.812. Reliance on the specified cut-off points was based on the original study and detailed as follows:

(A) The Knowledge of Influenza Scale encompassed 12 items. Responses of “Yes” were assigned 1 point, whereas “No” or “Don’t know” received 0, with an exception for questions 9 and 10, where “No” was valued at 1 point and “Yes” or “Don’t know” at 0. Consequently, the peak score achievable was 12. Respondents obtaining scores that exceeded the mean (6.8) were deemed to possess proficient knowledge, while those scoring below this threshold were categorized as having insufficient knowledge [13].

(B) The Adult Vaccine Hesitancy Scale (aVHS) consisted of 10 items with responses ranging on a 5-point Likert scale, from “strongly disagree” (5 points) to “strongly agree” (1 point). However, items 5, 9, and 10 deviated from this scoring pattern. The highest possible score was 50. Participants scoring within the range of 10–24 were classified as “Not exhibiting vaccine hesitancy,” whereas those within the 25–50 range were identified as vaccine-hesitant individuals [14].

For the analysis of variances between vaccine hesitancy, influenza knowledge, and baseline traits, both the Kruskal–Wallis and Mann–Whitney U tests were utilized. To evaluate the correlation between the assessed scales, Kendall’s tau-b test was employed. Furthermore, to pinpoint the factors (independent variables) correlated with a comprehensive understanding of influenza and hesitancy toward the annual influenza vaccine (dependent variables), logistic regression analyses were undertaken in two distinct models (unadjusted and adjusted for all covariates). Statistical significance was determined for *p*-values that were less than or equal to 0.05.

### 2.5. Ethical Considerations

The study was approved by the Alfaisal University Institutional Review Board (IRB log no. IRB-20222). It was explicitly clarified at the commencement of the survey that participation was voluntary and that, by proceeding, respondents were providing their implicit consent. The objectives of the study were also explained to the participants beforehand.

### 2.6. Representation across Demographics

The sample demographics gathered from the survey were contrasted with the demographics of the central region of Saudi Arabia and the nation at large to gauge its representativeness. While there were slight variances, these discrepancies are addressed and acknowledged in the discussion section of the paper, whereby the potential sampling bias inherent to web-based surveys is emphasized.

### 2.7. Supplementary Materials

Additional data tables supporting the results are available in the Supplementary Materials, and they are referred to as Supplementary Materials in the text.

## 3. Results

### 3.1. Baseline Characteristics

The study encompassed 589 responses from Saudi participants. Of these, 57% were female, 62.6% were married, and 37.9% were Saudi nationals. Over half (54.3%) were employed, and the majority (74.5%) did not have chronic diseases. Additional characteristics are presented in Table 1.

### 3.2. Responses to the aVHS

When asked about vaccine beliefs, 48.6% strongly believed in the essential role of vaccines for personal health, while 49.4% emphasized their importance for community health. However, 34.3% were unsure about the safety of newer vaccines compared to older ones. For a comprehensive breakdown, refer to Table 2.

### 3.3. Knowledge on Influenza and its Vaccine

Firstly, 89.8% agreed that flu infection is highly contagious, 69.1% granted that flu infection can cause hospital admission if severe, and 60.3% believed that the seasonal influenza vaccine could be given to people with chronic diseases. Moreover, 45.3% did not know if pregnant women should take the vaccine.

Additionally, 62.6% concurred that annual vaccination is the best way to protect from influenza, and 70.6% stated that seasonal vaccination is the best way to avoid complications. Participants who agreed that influenza vaccines are safe and effective were 66.2%, and 45.7% did not agree that seasonal vaccines weaken the immune system.

Furthermore, 55% did not know if the vaccines are recommended for children of more than 6 months of age. However, 52.8% declared that older people (65 years or above) should receive the influenza vaccine (Supplementary Table S1).

### 3.4. Inclination toward Influenza Vaccination

From our investigation, 34.3% of the subjects reported prior vaccination against influenza. Interestingly, an additional 26.1% expressed intentions to receive the vaccination within the study year. Nonetheless, a significant proportion, 39.6%, displayed reluctance toward the vaccine (Table 3).

### 3.5. Factors Influencing Influenza Vaccine Uptake

Table 4 shows the barriers to influenza vaccination. Predominantly, the notion of influenza as a benign ailment not necessitating vaccination emerged as the principal deterrent at 22.1%. Subsequent barriers encompassed forgetfulness (16%) and apprehensions about vaccine-associated adverse reactions (14.9%).

Table 5 catalogues the motivations prompting individuals to opt for influenza vaccination. The most prevalent inducements were physician recommendations (15.3%), occupational mandates (11.4%), and endorsements from acquaintances or kin (10%).

### 3.6. Quantifying the aVHS and Knowledge

As depicted in Supplementary Table S2, the mean score for the Adult Vaccine Hesitancy Scale (aVHS) was determined to be 22.87 out of a possible 50, with a standard deviation of 6.80. Simultaneously, the aggregate knowledge score concerning influenza settled at 6.81 out of a total 12, with a standard deviation of 3.21. Following the evaluation via the aVHS, the data revealed that 37.7% of the participants could be categorized as vaccine-hesitant. When it comes to general influenza awareness, a promising 56.7% showcased commendable knowledge, while 43.3% exhibited inadequate understanding (refer to Supplementary Table S3).

### 3.7. Correlation of the aVHS with the Participants’ Demographics

A comprehensive analysis was conducted to discern the relationship between the aVHS and the baseline characteristics of the participants. Interestingly, only nationality emerged as a significant predictor of vaccine hesitancy (*p*-value < 0.05). Specifically, 21.1% of the vaccine-hesitant group were non-Saudi nationals, as opposed to 16.6% being Saudis (Table 6).

### 3.8. Influenza Knowledge and Its Interplay with Demographic Factors

Analysis revealed that gender, age, marital status, employment status, and the presence of chronic diseases were not significant predictors of knowledge about influenza (all with *p*-values > 0.05). A noteworthy finding was the evident disparity in knowledge based on nationality: 38% of non-Saudi participants exhibited commendable knowledge, contrasted with 24.1% showcasing inadequate understanding (*p*-value = 0.004). In comparison, 18.7% of Saudi participants had a good grasp, while 19.2% displayed limited knowledge. Furthermore, the educational background was significantly associated with influenza knowledge (*p*-value = 0.00056), with the highest knowledge observed among postgraduate participants, followed by undergraduate and master’s degree holders (Table 7).

### 3.9. Variation in Mean Scores Based on Participant Characteristics

According to Table 8, a significant association was noted between the aVHS and two baseline variables (*p*-value < 0.05). Specifically, the participants within the 45–60 age bracket displayed superior knowledge, recording a mean score of 24.17 ± 7.01. Conversely, the 18–30 age cohort registered the lowest average score of 21.88 ± 6.96. Non-Saudi participants had a mean aVHS score of 22.08 ± 6.66, which was lower than their Saudi counterparts, who averaged 24.17 ± 6.85.

Upon the evaluation of influenza and vaccine knowledge of it, significant differences were observed across three characteristic variables (*p*-value< 0.05). Participants holding Ph. D. qualifications exhibited higher knowledge levels, averaging 8.34 ± 3.27, compared to other educational categories. Additionally, respondents without a Kingdom of Saudi Arabia nationality posted higher knowledge scores, averaging 7.28 ± 3.09. Among the employment categories, those currently employed exhibited the highest knowledge levels (Table 9).

### 3.10. Logistic Regression Analyses

Upon examination of the adjusted odds ratios in Table 10, most baseline characteristics did not exhibit a statistically significant association with the aVHS (*p*-value > 0.05). However, age proved an exception. Respondents aged 30–45 years exhibited significantly higher odds of vaccine hesitancy relative to the 18–30 age cohort (AOR 2.048, *p* = 0.036). Similarly, the age bracket of 45–60 years displayed 2.4 times elevated hesitancy odds in comparison with the 18–30 years group (AOR 2.399, *p* = 0.023). Furthermore, possessing a comprehensive understanding of influenza reduced the likelihood of hesitancy when juxtaposed against those with limited knowledge (AOR = 0.163, *p* < 0.001)

Table 11 illustrates that vaccine-hesitant respondents have significantly diminished odds of possessing an in-depth understanding of influenza relative to their non-hesitant counterparts (AOR 0.163, *p* < 0.001).

Table 12 reveals the non-significant relationships between most characteristics and the inclination to receive the vaccine (*p* > 0.05). However, the participants with chronic diseases demonstrated greater odds of vaccine acceptance (AOR = 3.635, *p* < 0.05) compared to their healthier peers. Conversely, vaccine-hesitant individuals and those with limited influenza knowledge were less likely to be vaccinated (AOR = 0.174, *p* < 0.001 and AOR = 5.096, *p* < 0.001, respectively).

Investigations into the relationships between vaccine hesitancy, knowledge of influenza, and the willingness to receive the influenza vaccine revealed significant correlations, as detailed in Table 13.

## 4. Discussion

The purpose of this study was to understand and analyze the level of hesitancy toward the influenza vaccine, as well as the myths and misconceptions that have arisen since the emergence of COVID-19. The World Health Organization (WHO) has identified vaccine hesitancy as one of the top ten health threats [15], thus making it crucial to understand and investigate the matter. Overall, we have anticipated a greater hesitancy surrounding the influenza vaccine among Saudi residents than in pre-COVID findings [16]. This is largely due to widespread rumors on various platforms and the pandemic’s impact on residents and their daily routines. Exposure to vaccination criticism and misinformation, often via social media and the Internet, plays a significant role in the crisis of vaccine hesitancy. While many people may not entirely trust the sources of these narratives, exposure to them could elicit emotions and bring about certain doubts and uncertainty [14,17]. However, while exposure to misinformation through social media platforms contributes to vaccine hesitancy, it is worth noting that social media can also serve as a vehicle for accurate information dissemination when used appropriately [18]. Several studies have highlighted the importance of strategic online health campaigns in shaping public perception positively [19,20].

It is important to recognize the different factors that play a role in the knowledge surrounding flu shots. Gender, age, and the presence of chronic disease were all found to be statistically insignificant factors (*p*-values > 0.05), while education level was found to be statistically significant (*p*-value = 0.00056). This can be shown by the fact that only 0.5% of respondents who had only completed primary school demonstrated good knowledge surrounding the influenza vaccine. Additionally, 6.3%, 6.6%, 7.5%, 10.2%, and 25.6% demonstrated good vaccine knowledge, with each number representing doctorate, master, high school, undergraduate, and postgraduate levels of education, respectively. Similarly, a study from the American Journal of Infection Control (AJIC) found that the lack of a high school education was one of the strongest predictors of vaccine hesitancy [21]. Another study also found that influenza vaccination rates were higher in adults with a bachelor’s degree or higher (45.1%) than in adults with less than a high school education (34.1%) [22]. Furthermore, participants with a strong knowledge of influenza tend to be 5.096 times more likely to receive the vaccine than those with poor knowledge (*p*-value = 0.000), which can be seen frequently when approaching vaccine hesitancy [23,24]. Overall, these findings are consistent with the available literature, and they suggest that an individual’s educational level is one of the strongest factors that can affect vaccine knowledge, which can thus impact a patient’s willingness to take the influenza vaccine. Despite the clear link between education level and vaccine hesitancy, it is essential to consider that the mere acquisition of formal education does not equate to a comprehensive understanding of vaccines. The quality and sources of the information available to the individuals is also relevant [25].

Concurrently, our results show that respondents who had a good knowledge of influenza were less likely to be resistant to the vaccine when compared to those with poor knowledge. Furthermore, the results also show that vaccine-hesitant respondents have an AOR of 0.163 for lower knowledge when compared to those who are not vaccine-hesitant (*p*-value = 0.000). This was also demonstrated in a study carried out in Austria for vaccine hesitancy, where the most common reason against vaccination was a “lack of specific knowledge” [26].

Varied educational backgrounds were obtained in this study, and it was found that participants with Ph. D. degrees were more knowledgeable than other education level subgroups. A higher degree of study was consistently seen to be associated with a higher agreeability to taking the influenza vaccine [27,28,29,30]. On the other hand, this could also be related to the finding that employed participants scored higher in terms of knowledge than others. A study in the Al-Jouf region in Saudi Arabia yielded comparable results, where employed respondents were more likely to receive the annual influenza vaccination than the unemployed respondents [31].

Further statistical analysis of the results found that respondents aged 30–45 had a hesitancy 2.048 times that of the 18–30 age group. In addition, it was also indicated that people aged 45–60 were 2.4 times more hesitant than those aged 18–30. This trend is also supported by a study conducted in Poland to assess the factors associated with attitudes toward the seasonal influenza vaccine after the COVID-19 pandemic, where the study’s results indicated that the odds of having a positive attitude toward seasonal influenza vaccination significantly increased after 60 years of age. This observation is consistent with the global trends in seasonal influenza vaccination uptake. Moreover, that study’s results reflected that, in the European Union/European Economic Area region, older age groups had a higher vaccination coverage rate compared to the youngest groups [32]. This can be explained by the fact that older individuals are considered a priority group for the seasonal influenza vaccination. Our study confirms that similar trends exist in Saudi Arabia, which is the direct result of the Ministry of Health’s promotion of influenza vaccination for high-priority groups, including the ages of 50 and older, as well as the awareness campaigns that increase this age group’s knowledge of the importance of vaccination [33]. In fact, a study conducted in Riyadh in 2022 showed that respondents who had a difference of being one year older were 1.6 times more likely to get the flu vaccine [23]. However, participants in the 45–60 age range had the greatest knowledge scores, while those in the 18–30 age range had the lowest. This can be compared to a study conducted in the United States that showed that, compared to older age groups, fewer young adults showed positive attitudes toward the influenza vaccine [34]. Yet, some studies have indicated varying reasons for hesitancy across age groups. For instance, younger adults may have concerns regarding vaccine side effects or the immediacy of the perceived threat, while older adults may have developed trust in vaccines over time, based on their prior exposure to vaccination campaigns [35].

As most people are unaware of the importance of the influenza vaccination, it is crucial to understand people’s attitudes toward the influenza vaccine after the COVID-19 pandemic. It was found that those who were vaccine-hesitant were less likely to take the vaccine when compared with non-hesitant respondents (*p*-value = 0.000). This was commonly seen at the beginning of the emergence of the COVID-19 pandemic, where the efficacy of vaccines was coated with misconceptions about how the vaccines work and the target population of the vaccines [36]. Further analysis revealed that individuals who have had chronic diseases were more willing to take the influenza vaccine than those who did not have any chronic diseases (*p*-value < 0.05). This could be due to the vaccine being particularly recommended to high-risk individuals rather than healthy adults by the Ministry of Health in Saudi Arabia [33], and these recommendations were issued since infection in these individuals could cause serious health conditions that could end up being fatal.

The majority of the study participants believed vaccines were important for their health. Conversely, only 34.4% of those surveyed said they were vaccinated for influenza. Healthcare workers frequently recognize a discrepancy between intention and action when it comes to individual health. This was evident when the survey responses revealed a moderate negative correlation between vaccine hesitancy and knowledge of influenza, as well as between vaccine hesitancy and a willingness to receive the vaccine (*p*-value < 0.05). Similar findings were shown in an Italian survey conducted in 2020, where most believed in the vaccine’s importance, yet remained unvaccinated due to the misconceptions and hesitance around it [37]. Out of the 588 responses of our surveyed participants, 386 of them reported that they have not taken the influenza vaccine yet or are not planning on taking it. The most chosen reasons out of the 13 listed ones were “Forgot” (*n* = 94 [16.0%]) and “I think flu is a simple disease and there is no need to prevent or vaccinate against it” (*n* = 130 [22.1%]). A study by Alabbad et al., conducted in 2016 in a hospital in Riyadh, showed similar results, where participants believed it “doesn’t have any positive effect or benefits” [16]. Another study conducted post-COVID in Jordan showed that the major reason for avoiding influenza vaccination was because “influenza was not considered as a threat” [12].

Trust in the source of information is an essential aspect that influences vaccine uptake, especially considering how the pandemic brought about extensive misinformation among everyone. This is also seen when surveyed participants were asked the question “Generally, I do what my doctor or healthcare provider recommends about vaccines for me,” where most of the respondents agreed with the statement (*n* = 506 [86.1%]). This notion is supported by a study that concluded that healthcare professionals are the most trusted source of vaccine-related information [38]. A moderately positive correlation was found between influenza knowledge and a willingness to receive the vaccine (*p*-value < 0.05). Furthermore, for the individuals who chose to be vaccinated with the influenza vaccine (*n* = 202 [34.4%]), most of them decided to go for the vaccine because of a doctor’s recommendation (*n* = 90 [15.3%]). This reinforces the fact that many rely on healthcare professionals to help them make important, life-altering decisions with respect to their health, and this includes vaccine uptake [39,40,41,42]. This was also seen when surveyed participants were asked the question “Generally, I do what my doctor or healthcare provider recommends about vaccines for me,” where most of the respondents agreed with the statement (*n* = 506 [86.1%]).

Regardless of the positive findings found above, this study nevertheless has certain limitations, such as the cross-sectional design and the online distribution of the questionnaire, which may have limited the representativeness of the study population. The collection of data online could have also introduced selection bias. Moreover, the online format of the survey may limit the study population to only those who have access to the Internet. Additionally, since the study was conducted in the central region of Saudi Arabia, other regions in Saudi Arabia were unfortunately not adequately represented. Furthermore, a particularly small number of respondents were over the age of 60; thus, that age group was not accurately represented.

## 5. Conclusions

This research identified a marked hesitancy among Saudi residents toward the influenza vaccine, one that was notably influenced by the aftereffects of the COVID-19 pandemic and the spread of misinformation on social media. These findings can furnish healthcare practitioners and policymakers with crucial insights that can be utilized to design strategies that boost vaccination uptake in the region. Public awareness campaigns need intensification, and they need to focus on the influenza vaccine’s safety and efficacy by drawing parallels with the COVID-19 vaccination success. Partnering with influential figures on social media can offer an effective avenue through which to reshape perceptions, given their significant reach to diverse demographics. The trust residents place in healthcare providers also suggests an opportunity. Platforms for these professionals to directly address concerns and queries related to vaccines can enhance public confidence. Concurrently, health authorities must utilize social media adeptly by rolling out informative campaigns, showcasing genuine testimonials, and presenting digestible scientific information to combat myths. To summarize, this study accentuates the necessity of countering vaccine hesitancy and misinformation, especially during pandemics. Adopting these evidence-informed strategies can enhance vaccine acceptance in Saudi Arabia, thereby promoting better health outcomes regionally and globally.

## Figures and Tables

**Table 1:** The baseline characteristics of the participants.

		Frequency	Percentage (%)
Gender	Male	253	43.0%
	Female	336	57.0%
Age	18–30	217	36.8%
	30–45	211	35.8%
	45–60	139	23.6%
	60<	22	3.7%
Education Level	Primary School	9	1.5%
	High School	81	13.8%
	Undergraduate	94	16.0%
	Postgraduate	301	51.1%
	Master	57	9.7%
	Doctorate	47	8.0%
Nationality	Non-Saudi	366	62.1%
	Saudi	223	37.9%
Marital Status	Single	220	37.4%
	Married	369	62.6%
Employment	Unemployed	231	39.2%
	Looking for a Job	23	3.9%
	Employed	320	54.3%
	Retired	15	2.5%
Presence of Chronic Disease	No	439	74.5%
	Yes	150	25.5%

**Table 2:** The aVHS questions.

		Frequency	Percentage (%)
1. Vaccines are important for my health	Strongly Disagree	16	2.7%
	Somewhat Disagree	13	2.2%
	Undecided	71	12.1%
	Somewhat Agree	203	34.5%
	Strongly Agree	286	48.6%
2. Vaccines are effective	Strongly Disagree	8	1.4%
	Somewhat Disagree	25	4.2%
	Undecided	83	14.1%
	Somewhat Agree	247	41.9%
	Strongly Agree	226	38.4%
3. Being vaccinated is important for the health of others in my community	Strongly Disagree	10	1.7%
	Somewhat Disagree	19	3.2%
	Undecided	60	10.2%
	Somewhat Agree	209	35.5%
	Strongly Agree	291	49.4%
4. All routine vaccinations recommended by the government are beneficial	Strongly Disagree	12	2.0%
	Somewhat Disagree	35	5.9%
	Undecided	93	15.8%
	Somewhat Agree	211	35.8%
	Strongly Agree	238	40.4%
5. New vaccines carry more risks than older vaccines	Strongly Disagree	39	6.6%
	Somewhat Disagree	101	17.1%
	Undecided	202	34.3%
	Somewhat Agree	152	25.8%
	Strongly Agree	95	16.1%
6. The information I receive about vaccines from the government is reliable and trustworthy	Strongly Disagree	13	2.2%
	Somewhat Disagree	27	4.6%
	Undecided	98	16.6%
	Somewhat Agree	229	38.9%
	Strongly Agree	222	37.7%
7. Getting vaccines is a good way to protect me from disease	Strongly Disagree	8	1.4%
	Somewhat Disagree	17	2.9%
	Undecided	74	12.6%
	Somewhat Agree	223	37.9%
	Strongly Agree	267	45.3%
8. Generally, I do what my doctor or healthcare provider recommends about vaccines for me	Strongly Disagree	11	1.9%
	Somewhat Disagree	17	2.9%
	Undecided	54	9.2%
	Somewhat Agree	258	43.8%
	Strongly Agree	249	42.3%
9. I am concerned about the serious adverse effects of vaccines	Strongly Disagree	24	4.1%
	Somewhat Disagree	72	12.2%
	Undecided	119	20.2%
	Somewhat Agree	209	35.5%
	Strongly Agree	165	28.0%
10. I do not need vaccines for diseases that are not common anymore	Strongly Disagree	73	12.4%
	Somewhat Disagree	147	25.0%
	Undecided	120	20.4%
	Somewhat Agree	157	26.7%
	Strongly Agree	92	15.6%

**Table 3:** The willingness to receive the influenza vaccine.

		Frequency	Percentage (%)
Willingness to Receive the Influenza Vaccine	I am not planning to take the vaccine	233	39.6%
	I am planning to take the vaccine this year	154	26.1%
	I have been vaccinated with the influenza vaccine	202	34.3%

**Table 4:** Barriers for influenza vaccination.

Reasons Why the Respondents Did Not Receive the Influenza Vaccine	Frequency	Percentage (%)
I don’t know where to go and get vaccinated	34	5.8%
Forgot	94	16.0%
I avoid medications	62	10.5%
I do not believe that the vaccine is effective	49	8.3%
I think the flu is a simple disease and there is no need to prevent or vaccinate against it	130	22.1%
The risk of acquiring the disease is low	41	7.0%
Fear of injection	28	4.8%
The Ministry of Health has not made vaccination obligatory	59	10.0%
The flu shot will make me sick	33	5.6%
I am concerned about the side effects	88	14.9%
I do not think that I belong to a group that vaccination is recommended for	43	7.3%
I had a bad experience with the previous flu vaccine	24	4.1%
I do not believe that the vaccine is safe	30	5.1%

**Table 5:** Reasons for influenza vaccination.

I Have Been Vaccinated Because…	Frequency	Percentage (%)
It was recommended by family/friend	59	10.0%
It was a job demand	67	11.4%
It was recommended by a doctor	90	15.3%
It was a Hajj requirement	18	3.1%
It was because of a media campaign	18	3.1%

**Table 6:** The Adult Vaccine Hesitancy Scale based on the baseline characteristics of the participants.

		Adult Vaccine Hesitancy Scale Categories				
		Non-Vaccine-Hesitant		Vaccine-Hesitant		*p*-Value
		Frequency	Percentage (%)	Frequency	Percentage (%)	
Gender	Male	167	28.4%	86	14.6%	0.107964
	Female	200	34.0%	136	23.1%	
Age	18–30	149	25.3%	68	11.5%	0.084950
	30–45	126	21.4%	85	14.4%	
	45–60	78	13.2%	61	10.4%	
	60<	14	2.4%	8	1.4%	
Education Level	Primary School	4	0.7%	5	0.8%	0.349932
	High School	57	9.7%	24	4.1%	
	Undergraduate	62	10.5%	32	5.4%	
	Postgraduate	178	30.2%	123	20.9%	
	Master	35	5.9%	22	3.7%	
	Doctorate	31	5.3%	16	2.7%	
Nationality	Non-Saudi	242	41.1%	124	21.1%	0.014477
	Saudi	125	21.2%	98	16.6%	
Marital Status	Single	142	24.1%	78	13.2%	0.387142
	Married	225	38.2%	144	24.4%	
Employment	Unemployed	150	25.5%	81	13.8%	0.070265
	Looking for a Job	12	2.0%	11	1.9%	
	Employed	200	34.0%	120	20.4%	
	Retired	5	0.8%	10	1.7%	
Presence of Chronic Disease	No	275	46.7%	164	27.8%	0.775175
	Yes	92	15.6%	58	9.8%	

**Table 7:** The knowledge of influenza and its vaccine based on the baseline characteristics of the participants.

		Knowledge of Influenza Categories				
		Poor		Good		*p*-Value
		Frequency	Percentage %	Frequency	Percentage %	
Gender	Male	102	17.3%	151	25.6%	0.205680
	Female	153	26.0%	183	31.1%	
Age	18–30	91	15.4%	126	21.4%	0.559287
	30–45	89	15.1%	122	20.7%	
	45–60	67	11.4%	72	12.2%	
	60<	8	1.4%	14	2.4%	
Education Level	Primary School	6	1.0%	3	0.5%	0.000561
	High School	37	6.3%	44	7.5%	
	Undergraduate	34	5.8%	60	10.2%	
	Postgraduate	150	25.5%	151	25.6%	
	Master	18	3.1%	39	6.6%	
	Doctorate	10	1.7%	37	6.3%	
Nationality	Non-Saudi	142	24.1%	224	38.0%	0.004784
	Saudi	113	19.2%	110	18.7%	
Marital Status	Single	94	16.0%	126	21.4%	0.830366
	Married	161	27.3%	208	35.3%	
Employment	Unemployed	109	18.5%	122	20.7%	0.079765
	Looking for a Job	10	1.7%	13	2.2%	
	Employed	126	21.4%	194	32.9%	
	Retired	10	1.7%	5	0.8%	
Presence of Chronic Disease	No	192	32.6%	247	41.9%	0.711077
	Yes	63	10.7%	87	14.8%	

**Table 8:** The difference between the mean of the Adult Vaccine Hesitancy Scale based on the baseline characteristics of the participants.

		Adult Vaccine Hesitancy Scale		
		Mean	Standard Deviation	*p*-Value
Gender	Male	22.79	6.96	0.503211
	Female	22.93	6.69	
Age	18–30	21.88	6.96	0.008589
	30–45	23.00	6.12	
	45–60	24.17	7.01	
	60<	23.14	8.90	
Education Level	Primary School	26.44	8.78	0.474148
	High School	22.07	6.36	
	Undergraduate	22.46	7.72	
	Postgraduate	23.10	6.56	
	Master	23.09	7.03	
	Doctorate	22.66	6.48	
Nationality	Non-Saudi	22.08	6.66	0.000143
	Saudi	24.17	6.85	
Marital Status	Single	22.48	7.03	0.138581
	Married	23.11	6.66	
Employment	Unemployed	22.49	6.80	0.073707
	Looking for a Job	23.78	5.77	
	Employed	22.87	6.75	
	Retired	27.27	8.28	
Presence of Chronic Disease	No	22.59	6.53	0.259970
	Yes	23.69	7.50	

**Table 9:** The difference between the mean of the Knowledge of Influenza Scale and its vaccine based on the baseline characteristics of the participants.

		Knowledge of Influenza		
		Mean	Standard Deviation	*p*-Value
Gender	Male	7.01	3.31	0.127296
	Female	6.67	3.13	
Age	18–30	6.88	2.88	0.631531
	30–45	6.82	3.37	
	45–60	6.60	3.32	
	60<	7.41	3.94	
Education Level	Primary School	5.44	2.24	0.000279
	High School	6.75	2.86	
	Undergraduate	7.04	2.89	
	Postgraduate	6.41	3.30	
	Master	7.65	3.29	
	Doctorate	8.34	3.27	
Nationality	Non-Saudi	7.28	3.09	0.000015
	Saudi	6.05	3.26	
Marital Status	Single	6.80	3.00	0.787038
	Married	6.83	3.33	
Employment	Unemployed	6.48	2.96	0.003566
	Looking for a Job	6.70	3.11	
	Employed	7.17	3.32	
	Retired	4.67	3.33	
Presence of Chronic Disease	No	6.73	3.18	0.258909
	Yes	7.05	3.28	

**Table 10:** Binary logistic regression between the baseline characteristics of the study population and the Adult Vaccine Hesitancy Scale.

		Adult Vaccine Hesitancy Scale							
				Confidence Interval 95%				Confidence Interval 95%	
		*p*-Value	COR	Lower	Upper	*p*-Value	AOR	Lower	Upper
Gender	Male	Ref.				Ref.			
	Female	0.108	1.320	0.941	1.854	0.091	1.431	0.945	2.169
Age	18–30	0.087	Ref.			0.135	Ref.		
	30–45	0.054	1.478	0.993	2.200	0.036	2.048	1.050	3.994
	45–60	0.017	1.714	1.103	2.663	0.023	2.399	1.126	5.114
	60<	0.630	1.252	0.502	3.126	0.252	2.081	0.594	7.295
Education Level	Primary School	0.359	Ref.			0.397	Ref.		
	High School	0.127	0.337	0.083	1.364	0.243	0.406	0.090	1.845
	Undergraduate	0.210	0.413	0.104	1.645	0.844	0.859	0.190	3.896
	Postgraduate	0.384	0.553	0.146	2.100	0.607	0.682	0.158	2.939
	Master	0.342	0.503	0.122	2.078	0.858	0.866	0.179	4.192
	Doctorate	0.231	0.413	0.097	1.754	0.807	0.816	0.160	4.167
Nationality	Non-Saudi	Ref.				Ref.			
	Saudi	0.015	1.530	1.087	2.153	0.137	1.366	0.906	2.058
Marital Status	Single	Ref.				Ref.			
	Married	0.387	1.165	0.824	1.648	0.244	0.688	0.366	1.291
Employment	Unemployed	0.092	Ref.			0.340	Ref.		
	Looking for a Job	0.229	1.698	.717	4.018	0.166	2.029	0.746	5.517
	Employed	0.558	1.111	.781	1.581	0.602	1.142	0.693	1.883
	Retired	0.020	3.704	1.224	11.205	0.193	2.434	0.637	9.301
Presence of Chronic Disease	No	Ref.				Ref.			
	Yes	0.775	1.057	0.722	1.548	0.803	0.943	0.595	1.495
Knowledge of Influenza Categories	Poor	Ref.				Ref.			
	Good	0.000	0.168	0.116	0.242	0.000	0.163	0.111	0.241

The derived logistic regression model was found to be statistically significant, as indicated by an X^2^ (16) = 122.53 with a *p*-value < 0.001. The Hosmer–Lemeshow test yielded a result of 12.14 with a *p*-value = 0.144718. The model’s efficacy in explaining the variance in the Adult Vaccine Hesitancy Scale was estimated at 25.6%, as gauged by the Nagelkerke R Square.

**Table 11:** Binary logistic regression of the baseline characteristics vs. influenza knowledge.

		Knowledge of Influenza and Its Vaccine							
				Confidence Interval 95%				Confidence Interval 95%	
		*p*-Value	COR	Lower	Upper	*p*-Value	AOR	Lower	Upper
Gender	Male	Ref.				Ref.			
	Female	0.206	0.808	0.581	1.124	0.972	0.993	0.663	1.486
Age	18–30	0.561	Ref.			0.638	Ref.		
	30–45	0.959	0.990	0.674	1.453	0.911	0.964	0.504	1.843
	45–60	0.246	0.776	0.506	1.191	0.327	0.688	0.326	1.453
	60<	0.614	1.264	0.509	3.138	0.797	0.850	0.246	2.939
Education Level	Primary School	0.001	Ref.			0.004	Ref.		
	High School	0.243	2.378	0.556	10.172	0.555	1.609	0.332	7.805
	Undergraduate	0.088	3.529	0.829	15.022	0.266	2.462	0.503	12.043
	Postgraduate	0.329	2.013	0.494	8.199	0.743	1.295	0.276	6.075
	Master	0.054	4.333	0.973	19.308	0.200	2.951	0.563	15.464
	Doctorate	0.011	7.400	1.567	34.935	0.064	5.125	0.910	28.872
Nationality	Non-Saudi	Ref.				Ref.			
	Saudi	0.005	1.620	1.158	2.268	0.083	0.700	0.468	1.048
Marital Status	Single	Ref.				Ref.			
	Married	0.830	1.038	0.741	1.454	0.947	1.021	0.554	1.880
Employment	Unemployed	0.089	Ref.			0.246	Ref.		
	Looking for a Job	0.734	1.161	0.490	2.756	0.231	1.826	0.682	4.890
	Employed	0.068	1.376	0.977	1.937	0.057	1.606	0.986	2.615
	Retired	0.153	0.447	0.148	1.348	0.561	1.482	0.393	5.586
Presence of Chronic Disease	No	Ref.				Ref.			
	Yes	0.711	0.932	0.640	1.356	0.873	0.963	0.608	1.526
Adult Vaccine Hesitancy Scale	Non-Vaccine-Hesitant	Ref.				Ref.			
	Vaccine-Hesitant	0.000	0.168	0.116	0.242	0.000	0.163	0.111	0.240

The logistic regression model was statistically significant, with an X^2^ (16) = 131.27 and a *p*-value < 0.001. Hosmer–Lemeshow test: 8.663781 (*p*-value = 0.37). The model explained 26.8% (Nagelkerke R Square) of the Knowledge of Influenza Scale.

**Table 12:** Binary logistic regression between the baseline characteristics of the study population and the willingness to receive the vaccine.

		Willingness to Receive the Vaccine							
				Confidence Interval 95%				Confidence Interval 95%	
		*p*-Value	COR	Lower	Upper	*p*-Value	AOR	Lower	Upper
Gender	Male	Ref.				Ref.			
	Female	0.387	0.863	0.617	1.206	0.140	1.406	0.895	2.211
Age	18–30	0.643	Ref.			0.569	Ref.		
	30–45	0.296	1.230	0.835	1.812	0.632	0.833	0.393	1.764
	45–60	0.597	1.124	0.729	1.734	0.213	0.581	0.248	1.365
	60<	0.340	1.577	0.618	4.024	0.435	0.573	0.141	2.324
Education Level	Primary School	0.115	Ref.			0.871	Ref.		
	High School	0.575	1.486	0.372	5.942	0.751	1.296	0.262	6.416
	Undergraduate	0.290	2.107	0.530	8.373	0.417	1.944	0.391	9.664
	Postgraduate	0.418	1.736	0.457	6.594	0.457	1.800	0.382	8.477
	Master	0.208	2.500	0.601	10.399	0.561	1.653	0.304	8.980
	Doctorate	0.062	4.091	0.933	17.936	0.413	2.115	0.352	12.727
Nationality	Non-Saudi	Ref.				Ref.			
	Saudi	0.004	1.655	1.178	2.323	0.208	0.749	0.477	1.175
Marital Status	Single	Ref.				Ref.			
	Married	0.037	0.697	0.496	0.979	0.085	1.863	0.919	3.780
Employment	Unemployed	0.011	Ref.			0.270	Ref.		
	Looking for a job	0.295	0.630	0.265	1.495	0.359	0.596	0.197	1.803
	Employed	0.007	1.607	1.135	2.276	0.152	1.494	0.863	2.587
	Retired	0.533	0.717	0.252	2.041	0.436	1.757	0.425	7.267
Presence of Chronic Disease	No	Ref.				Ref.			
	Yes	0.000	0.425	0.281	0.642	0.000	3.635	2.074	6.369
Adult Vaccine Hesitancy Scale	Non-Vaccine-Hesitant	Ref.				Ref.			
	Vaccine-Hesitant	0.000	0.135	0.093	0.197	0.000	0.174	0.112	0.271
Knowledge of Influenza Categories	Poor	Ref.				Ref.			
	Good	0.000	7.433	5.126	10.777	0.000	5.096	3.310	7.846

The derived logistic regression model was statistically robust, with an χ^2^ (17) = 226.04 and a *p* < 0.001. The Hosmer–Lemeshow test produced a value of 9.64 with a *p*-value of 0.29. This model elucidated on 43.1% of the variation in terms of willingness to receive the vaccine, as indicated by the Nagelkerke R Square.

**Table 13:** Kendall’s tau-b correlation between vaccine hesitancy, knowledge of influenza and its vaccine, and the willingness to receive the influenza vaccine.

			Adult Vaccine Hesitancy Scale Categories	Knowledge of Influenza Categories	Willingness to Receive the Vaccine or Have Already Received it
Kendall’s tau-b	Adult Vaccine Hesitancy Scale Categories	Correlation Coefficient	1.000	−0.409 **	−0.453 **
		*p*-value		0.000	0.000
	Knowledge of Influenza Categories	Correlation Coefficient	−0.409 **	1.000	0.456 **
		*p*-value	0.000		0.000
	Willingness to Receive the Vaccine or Have Already Received it	Correlation Coefficient	−0.453 **	0.456 **	1.000
		*p*-value	0.000	0.000	

** Indicates that the correlation is statistically significant at the 0.01 level (two-tailed).

## Data Availability

The data presented in this study are available on request from the corresponding authors.

## Supplementary Materials

The following supporting information can be downloaded at https://www.mdpi.com/article/10.3390/vaccines11101595/s1. Table S1: The questions of the knowledge of influenza and its vaccine; Table S2: The mean of Adult Vaccine Hesitancy Scale and the knowledge of influenza and its vaccine; Table S3: The categories of Adult Vaccine Hesitancy Scale and the knowledge of influenza and its vaccine.
