# Supplementary material for: Hesitance and Misconceptions about the Annual Influenza Vaccine among the Saudi Population Post-COVID-19
Source: Vaccines (Basel). 2023 Oct 15;11(10):1595. doi: 10.3390/vaccines11101595 (PMC10610791; doi:10.3390/vaccines11101595)
Supplement: Supplementary file 1 [file vaccines-11-01595-s001.zip › vaccines-2560019-supplementary.pdf]

Supplementary Tables

Table S1. The questions of the knowledge of Influenza and its Vaccine.

		Frequency	Percentage %
**1. Flu infection is highly contagious**	**No**	27	4.6%
	**I don't know**	33	5.6%
	**Yes**	529	89.8%
**2. Flu infection can sometimes be serious that a person must be admitted to the hospital**	**No**	78	13.2%
	**I don't know**	104	17.7%
	**Yes**	407	69.1%
**3. All pregnant women are advised to take the flu vaccine**	**No**	75	12.7%
	**I don't know**	267	45.3%
	**Yes**	247	41.9%
**4. Seasonal influenza vaccine is recommended to be given to all individuals with chronic disease**	**No**	63	10.7%
	**I don't know**	171	29.0%
	**Yes**	355	60.3%
**5. Annual vaccination is the best way to protect one from influenza**	**No**	126	21.4%
	**I don't know**	94	16.0%
	**Yes**	369	62.6%
**6. The best way to avoid the complications of influenza is by having seasonal vaccination**	**No**	65	11.0%
	**I don't know**	108	18.3%
	**Yes**	416	70.6%
**7. The influenza vaccine is safe and effective**	**No**	48	8.1%
	**I don't know**	151	25.6%
	**Yes**	390	66.2%
**8. Seasonal influenza vaccine is freely provided in every primary healthcare facility**	**No**	29	4.9%
	**I don't know**	212	36.0%
	**Yes**	348	59.1%
**9. Flu vaccine can cause influenza**	**No**	179	30.4%
	**I don't know**	210	35.7%
	**Yes**	200	34.0%
**10. Seasonal influenza vaccine weakens the immune system and renders one susceptible to infections**	**No**	269	45.7%
	**I don't know**	198	33.6%
	**Yes**	122	20.7%
**11. The influenza vaccine is recommended for children 6 months of age and above**	**No**	71	12.1%
	**I don't know**	324	55.0%
	**Yes**	194	32.9%
**12. The influenza vaccine is recommended for elderly individuals aged >65 years old**	**No**	51	8.7%
	**I don't know**	227	38.5%
	**Yes**	311	52.8%

Table S2. The Mean of Adult Vaccine Hesitancy Scale and the Knowledge of Influenza and its vaccine.

	Mean	Standard Deviation	Maximum	Minimum
**Adult Vaccine Hesitancy Scale**	22.87	6.80	50.00	10.00
**Knowledge of influenza**	6.81	3.21	12.00	.00

Table S3. The Categories of Adult Vaccine Hesitancy Scale and the Knowledge of Influenza and its vaccine.

		Frequency	Percentage %
**Adult Vaccine Hesitancy Scale Categories**	**Non-Vaccine Hesitant**	367	62.3%
	**Vaccine Hesitant**	222	37.7%
**Knowledge of influenza Categories**	**Poor**	255	43.3%
	**Good**	334	56.7%
